# Novel Combinatorial Strategy Using Thermal Inkjet Bioprinting, Chemotherapy, and Radiation on Human Breast Cancer Cells; an In-Vitro Cell Viability Assessment

**DOI:** 10.3390/ma14247864

**Published:** 2021-12-19

**Authors:** Aleli Campbell, Denisse A. Gutierrez, Colin Knight, Charlotte M. Vines, Rosalinda Heydarian, Alexander Philipovskiy, Armando Varela-Ramirez, Thomas Boland

**Affiliations:** 1 Metallurgical, Materials and Biomedical Engineering, The University of Texas at El Paso, El Paso, TX 79968, USA; Aleli.Campbell@ttuhsc.edu; 2 Border Biomedical Research Center, Department of Biological Sciences, The University of Texas at El Paso, El Paso, TX 79968, USA; dagutierrez5@utep.edu (D.A.G.); cdknight@utep.edu (C.K.); cvines@utep.edu (C.M.V.); avarela2@utep.edu (A.V.-R.); 3 Division of Hematology-Oncology, Department of Internal Medicine, The Texas Tech University Health Sciences Center El Paso, El Paso, TX 79968, USA; rosalinda.heydarian@ttuhsc.edu (R.H.); Alexander.Philipovskiy@ttuhsc.edu (A.P.)

**Keywords:** anticancer, bioprinting, cancer, chemotherapy, cytotoxicity, radiotherapy, thermal inkjet bioprinting

## Abstract

Background: Breast cancer (BC) continues to have the second highest mortality amongst women in the United States after lung cancer. For 2021, the American Cancer Association predicted 281,550 new invasive breast cancer cases besides 49,290 new cases of non-invasive breast cancer and 43,600 deaths from the metastatic disease. A treatment modality is radiation therapy, which is given for local control as well as palliation of patient symptoms. The initial step of new drug development is in-vitro cell studies, which help describe new drug properties and toxicities. However, these models are not optimal, and better ones have yet to be determined. This study uses bioprinting technology to elucidate the sensitivity of tumor cells to the combination of palbociclib (PD) and letrozole (Let) treatment. We hypothesize that this technology could serve as a model to predict treatment outcomes more efficiently. Methods: The breast cancer cell lines MCF7 and MDA-MB-231 as well as the normal breast epithelial cell line, MCF-10A, were treated with PD-Let with and without radiotherapy (RT), and cell viability was compared in pairwise fashion for thermally inkjet bioprinted (TIB) and manually seeded (MS) cells. Results: In absence of radiation, the TIB MCF7 cells have 2.5 times higher viability than manually seeded (MS) cells when treated with 100 µM palbociclib and 10 µM letrozole, a 36% higher viability when treated with 50 µM palbociclib and 10 µM letrozole, and an 8% higher viability when treated with 10 µM palbociclib and 10 µM letrozole. With 10 Gy of radiation, TIB cells had a 45% higher survival rate than MS cells at the lowest palbociclib concentration and a 29% higher survival rate at the intermediate palbociclib concentration. Without radiation treatment, at a concentration of 10 μM PD-Let, TIB MDA-MB-231 cells show a 8% higher viability than MS cells when treated with 10 µM PD and 10 µM Let; at higher drug concentrations, the differences disappeared, but some 1.7% of the TIB MDA-MB-231 cells survived exposure to 150 μM of PD + 10 μM letrozole vs. none of the MS cells. These cells are more radiation sensitive than the other cell lines tested and less sensitive to the combo drug treatments. We observed an 18% higher survival of TIB MCF-10A cells without radiation treatment when exposed to 10 μM PD + 10 μM Let but no difference in cell survival between the two groups when radiation was applied. Independent of growth conditions, TIB cells did not show more resistance to radiation treatment than MS cells, but a higher resistance to the combo treatment was observed, which was most pronounced in the MCF-7 cell line. Conclusion: Based on these results, we suggest that TIB used in in-vitro models could be a feasible strategy to develop and/or test new anticancer drugs.

## 1. Introduction

Breast cancer (BC) is the second leading cause of cancer death in women after lung cancer in the United States. For 2021, the American Cancer Association predicts 281,550 new invasive breast cancer cases besides 49,290 new cases of non-invasive breast cancer and 43,600 deaths from the metastatic disease, the incurable and lethal stage of BC. The introduction in 2015 of CDK4/6 inhibitors, such as palbociclib, ribociclib, and abemociclib, combined with anti-estrogen therapy into the standard of care of afflicted patients led to statistically significant improvement of survival [1,2,3].

Most commonly, hormone receptor-positive BC metastasizes to bones, increasing the risk of bone fractures, which cause significant pain. Palliative radiation therapy (RT) helps achieve rapid pain control and prevent further bone destruction. In the majority of cases, systemic therapy is withheld during RT. However, the safety and tolerability of concurrent RT with palbociclib-letrozole (PD-Let) has only been tested in few small clinical trials [4,5,6]. Adverse effects noted from this study were similar to those reported in the PALOMA trial [7]. No skin toxicities were reported; digestive toxicities were described to grade I and grade II mucositis; hematologic toxicities reported were grade III neutropenia, anemia, and thrombocytopenia. All subjects in those trials reported good symptom control. However, there is a lack of in-vitro cell studies of the effect of PD-Let treatment concurrent with radiation therapy (RT), which would help discover new drug properties and toxicities, and the optimal model for such an in-vitro study has not been determined. Ideally, such a model would employ the most resistant cells, which will survive even high doses of the combination treatment.

Thermal inkjet bioprinting (TIB) is a novel approach used to bioprint biomaterials or cells to mimic tissue or organoid structures in vitro [8,9,10]. In a previous study, we identified significant RNA differences between thermally inkjet bioprinted and manually seeded MCF-7 breast cancer cells [11]. We found that TIB MCF7 cells activate many analytes, including RSK1/2, HSP27, p38δ/β/γ, MSK2, p53, MKK3/6, TOR, p38, and JNK. In an RNA sequential analysis, we observed the following five genes differentially expressed NRN1L, LUCAT1, IL6, CCL26, and LOC401585 amongst hundreds of other differentially expressed genes that have been associated with cell survival and the suppression of apoptosis. These data indicated that TIB causes large-scale gene alterations when compared to the manually seeded cells. Thus, this strategy to develop tumor models is encouraging and warrants further investigation.

To the best of our knowledge, the combination therapy of PD-Let with RT has not been evaluated in either TIB or normally manually seeded (MS) cancer cells. In-vitro cell testing is one of the early steps in the process of drug discovery. Yet, in many cases, the results do not recapitulate in-vivo settings due to complicated reactions typical of in-vivo environments [12]. Recently, several authors have used 3D bioprinting to construct improved in-vitro models [13,14]. We propose here that thermal inkjet bioprinting is a promising method to develop in-vitro tumors for further exploration of drug discovery. These tactics may be ideal for drug discovery because they could be used to better understanding of drug resistance therapies for new and approved drugs [15]. Therefore, we decided to explore this technique further in breast cancer cells in vitro with approved breast cancer drugs, the combination of PD-Let, and incorporated RT.

## 2. Materials and Methods

### 2.1. Drug Preparation and CC_50_ Determination

**Figure 1:**
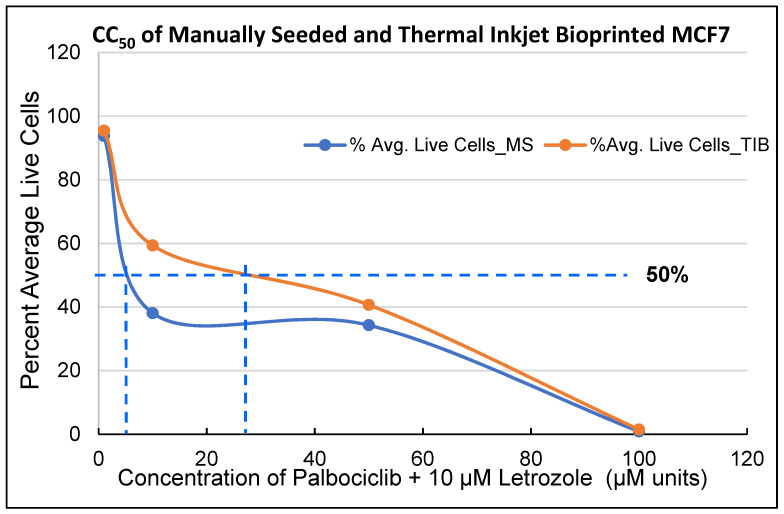
Cytotoxic concentration-50% (CC_50_) of Manually Seeded Cells and Thermal Inkjet Bioprinted MCF7 Cells, 25 h post drug exposure. CC_50_ obtained for TIB 29.9 µM PD plus 10 µM letrozole and for the MS cells 7.9 µM PD plus 10 µM letrozole (see summary table with CC_50_ values for all three cell lines that were tested). CC_50_ values compared are for manually seeded and thermal inkjet bioprinted cells.

PD 0332991 HCL (Palbociclib) and letrozole (Let; Formula: C_17_H_11_N_5_) were obtained from Fisher Scientific (Waltham, MA, USA). Palbociclib was dissolved in dimethyl sulfoxide (DMSO) to obtain a stock solution of 1 mM. Letrozole was dissolved in DMSO as a 100 mM stock solution. Drugs were serially diluted to determine the cytotoxic concentration 50% (CC_50_) of the TIB and manually seeded cell samples required to kill 50% of the cell population [16,17]. Additionally, the CC_50_ values were determined by using the cytotoxicity values obtained experimentally and the linear interpolator webpage (https://www.johndcook.com/interpolator.html (accessed on 16 December 2021)) as previously described [18]. This assay was conducted three times independently in 4–5 wells each time to determine reproducibility for both TIB and MS cells at 1, 10, 50, and 100 µM PD + 10 µM letrozole; graphs and CC_50_ values obtained are shown in Figure 1. TIB and MS MCF7 cells were incubated for 24 h prior to drug exposure. The same procedure explained in Section 2.5 was followed to determine cell viability.

### 2.2. Cell Culture

Three cell lines were utilized in this investigation. The MCF7 (ATCC^®^ HTB-22^™^, Manassas, VA, USA) BCCs were maintained in Eagle’s minimum essential medium (EMEM) supplemented with 0.01 mg/L human recombinant insulin and 10% fetal bovine serum (FBS). The MDA-MB-231 BCCs were cultured in the complete growth medium, Dulbecco’s modification of Eagle’s medium (DMEM) supplemented with 10% FBS, and 1% antibiotic/antimycotic solution, and the non-tumorigenic MCF-10A breast cells were kept in DMEM/F-12 medium supplemented with 10% FBS, 1% penicillin-streptomycin solution, 100 µg/mL epidermal growth factor (EGF), 0.5 µg/mL hydrocortisone, and 10 µg/mL of human recombinant insulin. Trypsin w/EDTA 0.25%, and sterile phosphate-buffered saline (PBS) solution were used to passage the cells. Cells were incubated as previously detailed [11,17]. Briefly, MCF7s, MDA-MB-231, and MCF-10As were cultured per their respective cell protocol in a humidified incubator maintained at 37 °C with 5% CO_2_ atmosphere until 80–90% confluency was reached. In preparation for bioprinting, the cells were rinsed with PBS, detached with trypsin, and counted using trypan blue and a hemocytometer.

### 2.3. Bioprinting Process

Approximately, 1.2 × 10^6^ cells/mL were resuspended in 1x PBS and used as the bioink cell solution. Modified inkjet cartridges were sonicated in deionized water for 15 min and tested in a previously modified thermal inkjet printer (HP Deskjet 340 Printer, HP, Palo Alto, CA, USA) inside of a biological safety cabinet [19,20]. Next, 100 µL of cell solution was added to a modified sterile inkjet printer cartridge [21]. The cell solution was bioprinted over one-half of the 96-well plate containing 200 µL of pre-incubated media [11]. Manually seeded cells (MS) were added to the other half of the 96-well plate. Both TIB and MS cells were incubated immediately after with two media changes first at three days post bioprinting and then prior to the drug treatments.

### 2.4. Concurrent Treatment with Palbociclib, Letrozole, and Radiotherapy

Thermal inkjet bioprinted and manually seeded MCF7 cells were incubated for 5 days post bioprinting with two media changes to allow cells to settle and grow in their new environment. On the 5th day, each well-containing cell was adjusted to 10, 50, 100, and 150 µM PD and 10 µM Let. Letrozole remained constant for all treatments at 10 µM and is referred to as ##.# µM PD-Let. After 12 h post inoculation then at 24-h intervals for five consecutive days, all PD-Let-treated and non-treated cells were exposed to fractionated irradiation (RT) of 2, 4 (twice), and 5 (twice) Gray (Gy) for a cumulative dose of 10 and 20 Gy [22]. Cell media was replenished after cumulative irradiation exposure of 10 Gy.

### 2.5. Cytotoxicity Quantification

Cytotoxicity was assessed using an automated live-cell differential nuclear staining (DNS) imaging assay. The MDA-MB-321 cells were incubated for 2 days post bioprinting with one media change (higher confluency was observed on these cells post-bioprinting); the same drug concentrations as the MCF7 cells was used. Cytotoxicity was evaluated 12 h post irradiation by using the live-cell differential nuclear staining (DNA) assay utilizing Hoechst 33342 and propidium iodide (PI) stains as previously described [16]. The DNS assay has been confirmed to be suitable, robust, reliable, and also washing-step free, efficient for both primary and secondary screens of promising cytotoxic compounds [16,17]. Images of cells from individual wells used to quantify their viability profiles were independently captured by using the IN Cell Analyzer 2000 multi-well plate reader bioimager system (GE Healthcare, Systems, Chicago, IL, USA). To examine suitable numbers of regions of interest (ROIs; corresponding to the nuclei/cell numbers), 2 × 2 montages from four contiguous image fields were acquired per well using a 10× objective [17]. Next, for image analysis and segmentation purposes, based on Hoechst and PI nuclear staining, discriminating the live/dead cells, the IN Cell Analyzer 2000 workstation 3.2 software was utilized (GE Healthcare) [16,23].

### 2.6. Statistical Analysis

Experimental data points as well as all controls were done using at least three independent measurements. Averages and their corresponding standard deviations (±) are reported to denote the experimental variability. Statistical relevance between two samples was determined by using the two-sample *t*-tests and/or ANOVA tests, where appropriately applicable. *p*-Values of ≤0.05 were deemed statistically significant.

## 3. Results

Thermal inkjet bioprinted cells were previously analyzed and explained in great detail by Campbell et al. [11]. Viability, apoptosis, phosphorylation, and RNA sequence of the bioprinted cells were compared to the manually seeded cells. Briefly, amongst the key finding from the phosphorylation assays, we found the deactivation of GSK-3β via phosphorylation by the Akt/PKB pathway, thus inhibiting cells from entering apoptosis. Therefore, the inhibition of drug-induced apoptosis may be triggered by GSK-3β, and therefore, TIB cells show greater survival. In addition, phosphorylation of heat shock proteins were observed in bioprinted cells, and these increased levels may enhance cell survival/resistance in cancer cells. P38 MAPKs were also highly phosphorylated, and this activation has been linked to tumor cell survival. MSK2 was phosphorylated in bioprinted cells, and this is putatively assisting regulation of glycogen metabolism, which is key to cell survival of chemotherapeutic treatments, as glucose is largely metabolized by glycolysis in cancer cells. With respect to RNA seq analysis, the most striking change in expression levels between bioprinted and manually seeded cells was the 350-fold increase in cytochrome C genes, which are involved in activating and metabolizing several chemotherapeutic drugs and compounds widely used in pharmacotherapy. Since all these molecular changes upon bioprinting tend to favor cell survival upon chemotherapeutic drug treatment, we report these results next.

### 3.1. Palbociclib-Letrozole and Radiotherapy Treatment of Thermal Inkjet Bioprinted and Manually Seeded MCF7 Breast Cancer Cells (BCCs)

Each experiment was repeated three times with N = 4–5 duplicates each; thus, we present here data on 12–15 individual experiments per control or treatment group. The CC_50_ values obtained for TIB compared to MS cells was approximately four times higher (~29 µM PD Vs ~8 µM PD) see Figure 1, Table 1. Overall, CC_50_ values for MS cells are lower for all three cell lines tested, which further confirms our hypothesis that TIB cells are more resilient that manually seeded cells. Calculations are also included when a radiation of 10 Gray was added to the drug treatment.

**Figure 2:**
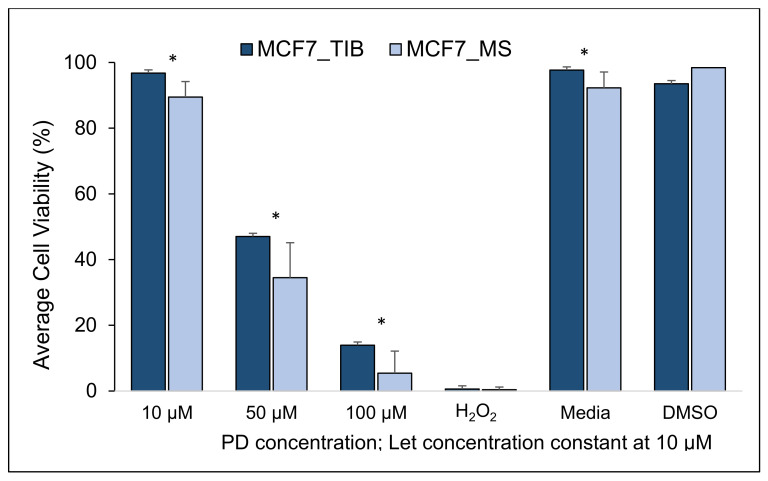
Cytotoxic effect of PD-Let on TIB and MS MCF7 breast cancer cells. No RT exposure (RT = 0). (* indicates statistically significant with a *p*-value ≤ 0.05). TIB, thermal inkjet bioprinted, MS, manually seeded.

**Figure 3:**
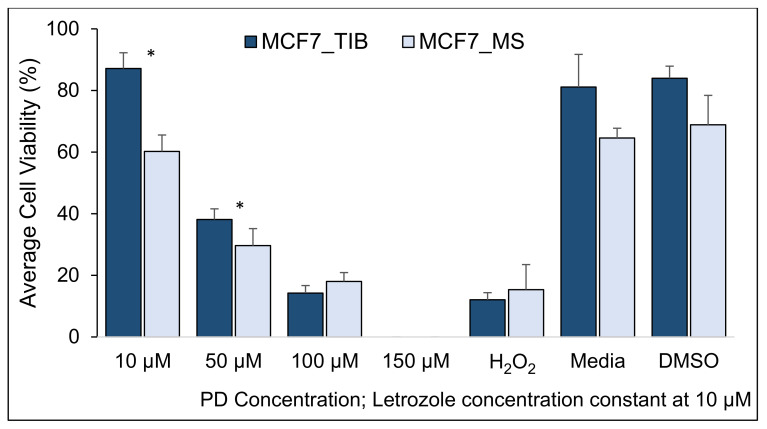
Cytotoxic effect of PD-Let concurrently with RT on TIB and MS MCF7 breast cancer cells. RT exposure was 10 Gy applied in 3 fractions (* indicates statistically significant with a *p*-value ≤ 0.05).

**Figure 4:**
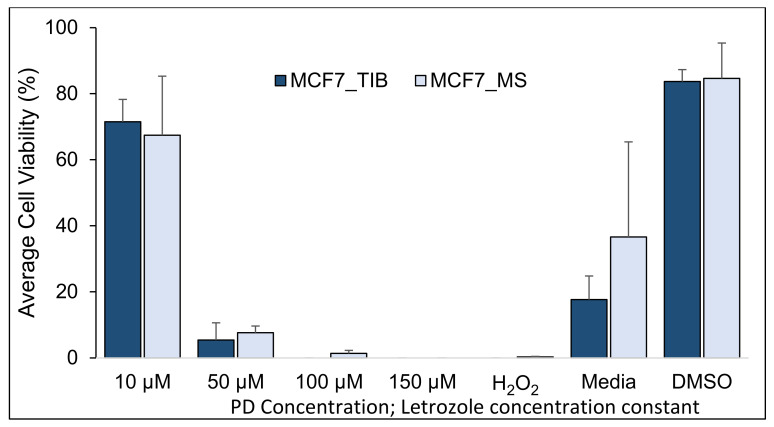
Cytotoxic effect of PD-Let concomitantly with RT on TIB and MS MCF7 breast cancer cells. RT applied was 20 Gy in 5 fractions.

**Figure 5:**
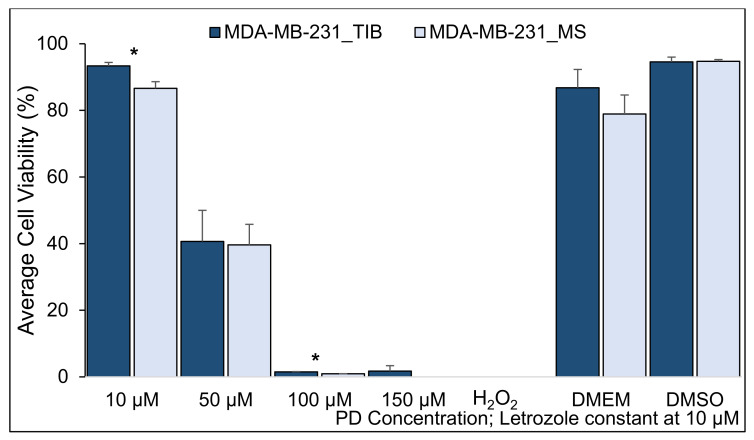
Cytotoxic effect of PD-Let on TIB and MS MDA-MB-231 breast cancer cells. No RT was applied (RT = 0). Average cell viability of TIB and MS MDA-MB-231 basal breast cancer cells (* indicates statistically significant with a *p*-value ≤ 0.05).

To determine whether TIB affects the ability of BCCs to survive chemotherapy ± RT, we compared TIB to MS in MCF7 “luminal type A” estrogen receptor-positive (ER+), progesterone receptor-positive (PR+), and human epidermal growth factor receptor 2 negatives (HER_2_^−^) cells. The average cell viability from TIB and MS MCF7 breast cancer cells after treatment with PD and Let without RT are summarized in Figure 2, Figure 3, Figure 4 and Figure 5 and Table 2, Table 3 and Table 4. The viability of bioprinted MCF7 BCCs exposed to 10 µM PD-Let without RT was 96.8% ± 1.9, whereas for the manually seeded cells, the cell viability average was 89.5% ± 4.7 (*p* < 0.001); see Table 2, Table 3 and Table 4 and Table 11 for overall results. To examine the effect of stress, we exposed the cells to H_2_O_2_, where the average cell viability of TIB and MS cells was not significantly different at 0.6% ± 0.5 and 0.4% ± 0.8, respectively (*p* = 0.5).

As mentioned above, palbociclib plus letrozole are often used either before or after treatment with radiation (RT). Therefore, to determine if bioprinting affected cell viability with the addition of RT, we evaluated palbociclib plus letrozole combined with RT at 0, 10, and 20 Gray (Gy). In the absence of RT, for all concentrations of PD-Let tested, the TIB cells survived at statistically significantly higher percentages, as shown in Figure 2. The TIB MCF7 cells have 2.5 times higher viability than manually seeded (MS) cells when treated with 100 µM palbociclib and 10 µM letrozole, a 36% higher viability when treated with 50 µM palbociclib and 10 µM letrozole, and an 8% higher viability when treated with 10 µM palbociclib and 10 µM letrozole, all significantly higher (*p* < 0.03), see Table 1.

The survival of TIB and MS MCF7 BCCs after treatment with PD-Let given concomitantly with RT of 10 Gy in three fractions is shown in Figure 3. At concentrations of 10 µM palbociclib and 10 µM letrozole, the average cell viability of TIB cells was 87% ± 5.15, while for the manually seeded cells, it was 60% ± 5.3 (*p* < 0.05). Moreover, at concentrations of 50 µM palbociclib and 10 µM letrozole, the average cell viability of TIB cells was 47% ± 8.6, while for the manually seeded cells, it was 34% ± 10.7 (*p* < 0.05). For the highest palbociclib treatment, the respective survival was 14% and 18%. Thus, TIB cells had a 45% higher survival rate than MS cells at the lowest palbociclib concentration and a 29% higher survival rate at the intermediate palbociclib concentration. At the highest palbociclib concentration, a somewhat lower survival rate is observed, but this is not statistically significant at the 0.05 level (see Table 2). At the highest concentration of PD (150 μM), the mean cell viability was not evaluated due to cell fragmentation.

We increased the radiation dose to 20 Gy and found that when TIB and MS MCF7 BCCs were treated with 10 μM PD + 10 μM Let concurrently with RT of 20 Gy given in five fractions, the TIB cells had a significant increase in cell viability at 71% ± 6.8 when compared to MS cells 67% ± 18.0, as shown in Figure 4 and Table 4. BCCs treated with 50 μM PD + 10 μM Let concurrently with RT of 20 Gy given in five fractions showed 5% ± 5.2, and 8% ± 2.1 survival TIB vs. MS, respectively. None of the differences observed between of TIB vs. MS BCC survival rates that received the 20 Gy radiation were statistically significant.

### 3.2. Palbociclib-Letrozole and Radiotherapy Treatment of Thermal Inkjet Bioprinted and Manually Seeded MDA-MB-231 Breast Cancer Cells

Since TIB prevented the killing of the luminal BCC line MCF7, we questioned whether TIB would have a similar effect on the more aggressive Claudin-low type MDA-MB-231 (ER-, PR-, Her2-) triple-negative BCCs. We then compared the viability of the TIB and MS MDA-MB-231 cell line with respect to PD-Let, both without and with radiation.

Without radiation treatment, at a concentration of 10 μM PD-Let, similar to what we observed in TIB MCF7 cells, cell viability was 93.4% ± 1.05 for the TIB samples, while it was 86.6% ± 2.0 for MS cells (*p* < 0.05), as shown in Figure 5 and Table 5. When we increased the dose to 50 μM PD + 10 μM Let, TIB to MS cell survival was 40.6% ± 9.3 TIB vs. 39.6% ± 6.2 MS; there was no significant difference. However, at 50 μM PD + 10 μM Let, the average survival of the TIB and MS cells was 1.5% ± 0.1 and 0.9% ± 0.05, respectively, which was statistically significant (*p* = 0.003). Some 1.7% ± 1.7 the TIB cells even survived exposure to 150 μM of PD + 10 μM Let, a concentration that killed all MS cells. Clearly, the TIB is affecting a few of these cells, enabling survival.

**Figure 6:**
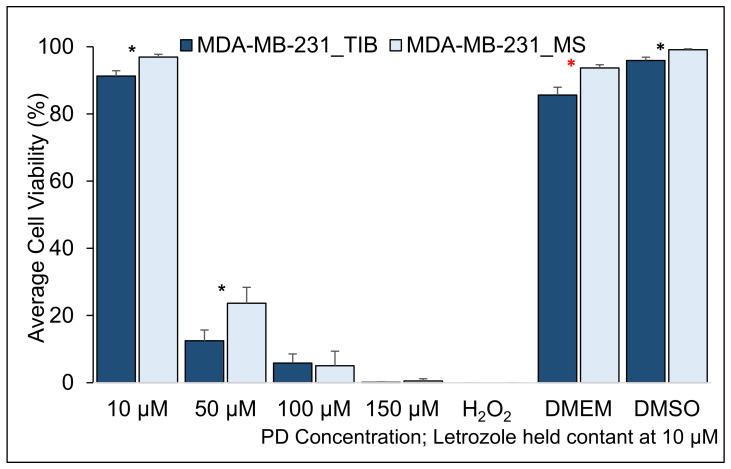
Cytotoxic effect of PD-Let with a total RT of 10 Gy on TIB and MS MDA-MB-231 breast cancer cells. RT of 10 Gy given in 3 fractions. Average cell viability of TIB and MS MDA-MB-231 basal breast cancer cells (* indicates statistically significant with a *p*-value ≤ 0.05).

The effect of 10 Gray RT given in three fractions on the survival of the Claudin-low MDA-MB-231 cells is shown in Figure 6 and Table 6. We found significantly lower cell survival of TIB cells compared to the MS cells for both low and intermediate PD concentrations. This effect disappeared for the higher PD concentrations of 100 and 150 μM. This indicates that the radiation renders the TIB MDA-MB-231 cells more susceptible to the drug treatments at low doses. However, this sensitivity is not seen at higher PD concentrations, also indicating that some cells will survive the combination treatment.

**Figure 7:**
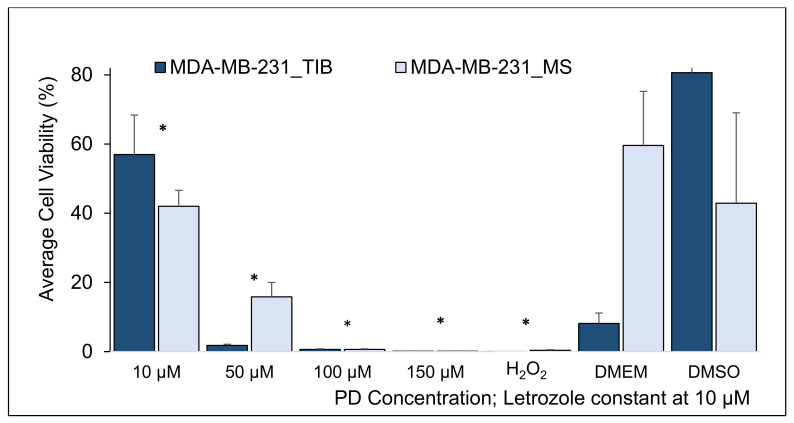
Cytotoxic effect of PD-Let with total RT of 20 Gy on TIB and MS MDA-MB-231 breast cancer cells. RT applied in 5 fractions. Average cell viability of TIB and MS MDA-MB-231 basal breast cancer cells (* indicates statistically significant with a *p*-value ≤ 0.05).

Next, we increased the radiation dose to 20 Gy and found that at 10 μM PD-Let, 57% ± 11.4 TIB cells remained viable in contrast to 42% ± 4.6 MS cells, as shown in Figure 7 and Table 7. At the higher PD dose of 50 μM PD-Let, 1.8% ± 0.4 TIB remained viable, while 16% ± 4.2 MS cells were alive at the end of the assay. At the increased dose of 100 μM PD-Let, 0.6% ± 2.7 TIB and 51.8% ± 4.3 MS cells survived. Looking at the effect of radiation in addition to the PD-Let, the higher doses do render the treatments more effective, killing more TIB cells, something that is only observed for the highest radiation dose for MS cells. However, no treatment killed all cells.

### 3.3. Palbociclib-Letrozole and Radiotherapy Treatment of Thermal Inkjet Bioprinted and Manually Seeded MCF-10A Breast Cells

**Figure 8:**
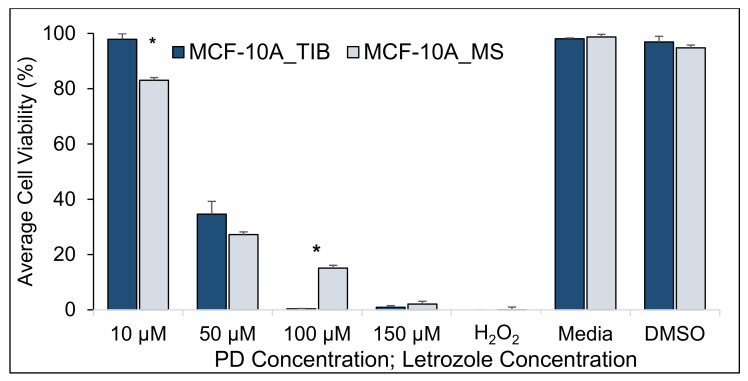
Average cell viability of TIB and MS MCF-10A non-tumorigenic breast cells treated with PD-Let. Cytotoxic effect of palbociclib + letrozole in parallel with RT = 0 (* indicates statistically significant with a *p*-value ≤ 0.05).

**Figure 9:**
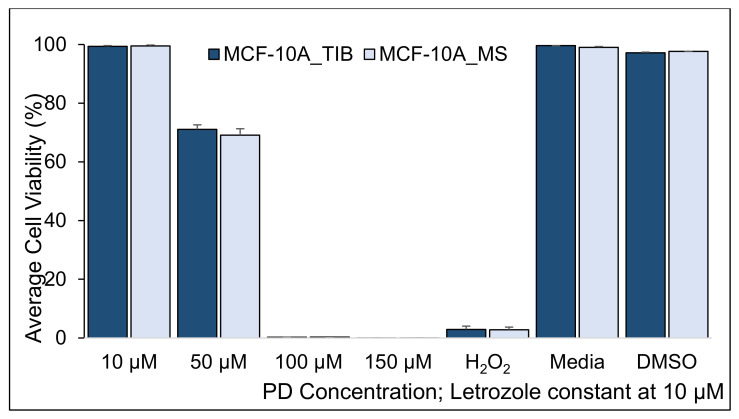
Average cell viability of TIB and MS MCF-10A, non-tumorigenic breast cells, treated with PD-Let in parallel with RT. Cytotoxic effect of palbociclib + letrozole and RT of 10 Gy on TIB and MS MCF-10A non-tumorigenic breast cells.

**Figure 10:**
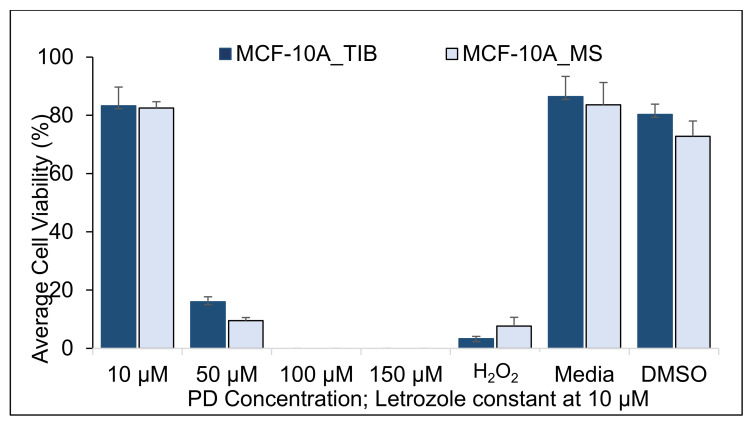
Average cell viability of TIB and MS MCF-10A non-tumorigenic breast cells treated with PD-Let in parallel with RT of 20 Gy given in 5 fractions. Cytotoxic effect of palbociclib + letrozole in parallel with RT on TIB and MS MCF-10A non-tumorigenic breast cells.

Since it was clear that bioprinting affects the viability of breast cancer cells in the presence of drugs and radiation, we investigated the effect of bioprinting on non-cancerous breast cells, the MCF-10A line. Figure 8, Figure 9 and Figure 10 and Table 8, Table 9 and Table 10 show the viability of TIB and MS MCF-10A cells after concurrent PD-Let and RT of 0, 10, and 20 Gy treatments.

We detected an 18% higher survival of TIB cells without radiation treatment when exposed to 10 μM PD + 10 μM Let (*p* = 0.03). This difference disappears for 50 μM PD + 10 μM Let treatment and reverses for 100 μM PD + 10 μM Let treatment. At 150 μM PD + 10 μM Let, no significant differences are observed. When radiation is added to the treatment, we observe no significant differences in survival rates between TIB and MS cells for all radiations and drug concentrations that were tested. We hypothesize that the TIB will also affect apoptotic behavior for non-cancerous cells but that the radiation treatment negates those effects. In other words, we do not necessarily select more resistant cells by bioprinting normal cells. In contrast, we select more aggressive cells that are more difficult to treat after bioprinting cancer cells. At present, we have no explanation on why MS MCF-10A shows such high viability when treated with 100 μM PD + 10 μM Let.

## 4. Discussion

As observed in the CC_50_ experiment, MCF7 TIB cells displayed higher resistance to PD plus letrozole as compared to the MS cells (8 µM vs. 30 µM), but this difference in the CC_50_ values disappears at high radiation. These results were expected, as we observed a similar trend in previous experiments when TIB and MS cells were exposed to tamoxifen [21] and fulvestrant (not included in this report). We also note an increase in PD CC50 values for low radiation exposure (at 10 Gy) for both MCF-7s and MCF-10As whether bioprinted or not. These data indicate that these cells become more resistant to the drug combination after low radiation. This resistance disappears for higher radiation exposure. X-radiation-related decline in EGF-induced DNA synthesis has been described before [24], and this could be the case that this decline also renders the drug treatment less effective. As also discussed later, the MDA-MB-231 cells displayed low sensitivity to PD compared to the MCF-7s. This is also seen in the CC50 values. However, the MDA-MB-231 are more sensitive to radiation than the MCF-7s judging from the CC_50_ data in Table 1. In addition, the MDA-MB-231 are about as sensitive to the drugs as the non-cancer cells when no radiation is applied, which is why these drugs are not used in triple-negative cancers.

Preclinical data indicate that the PD’s combination with RT has ameliorative effects in cancer therapies [15,25]. It was demonstrated that the CDK 4/6 inhibitors suppress downstream signaling pathways, causing cell cycle arrest at the G1-S phases [1,26]. In theory, sensitivity to radiotherapy depends upon the cell cycle, with cells in G1/S being the most sensitive to radiation [27,28]. In an analysis where 47 cell types were included, Finn et al. [1] demonstrated that the efficacy of palbociclib is more conspicuous in luminal cell types, including MCF-10A and MCF7 cells. In contrast, MDA-MB-231 cells displayed low sensitivity to PD, which was also recorded from the results obtained here.

We noted that at low PD-Let concentrations, MCF-10A cells have higher viability when exposed to 10 Gy than when RT was omitted; at this point, we do not have an explanation for this phenomenon although we ruled out the breakdown of PD and Let due to radiation, as we would have observed this in all samples exposed to radiation. Thermal Inkjet Bioprinted MCF-10A cells have a 22% higher viability than the manually seeded cells when treated with 50 µM PD + 10 µM Let and RT of 20 Gy.

In the TIB, MDA-MB-231 cells displayed an 8% higher viability than MS cells when treated with 10 µM PD-Let without radiotherapy. That difference disappeared with 10Gy radiation but was increased to 35% higher survival at 20 Gy although the absolute survival was lower for both MS and TIB cells at this radiation. Both TIB and MS MDA-MB-231 cells were initially sensitive to the combined therapy when a loss of cell proliferation was observed; however, more than 50% of the cell population survived both systemic and radiation therapies at 10-µM drug concentrations. In fact, we observed that TIB MDA-MB-231 cells at the lower concentrations of PD (10 µM and 50 µM) in parallel with RT survived 48 h post treatment. Additionally, the cells that were exposed to the solvent control only exhibited a similar survival rate, suggesting that the RT is not causing significant toxicity at this dose tested. This effect is expected, as it has been reported that PD affects the cyclin D-Retinoblastoma (Rb) pathway in cancer cells that express the Rb gene, and triple-negative breast cancer cells have been associated with loss of Rb expression [29,30].

TIB and MS MDA-MB-231 cells were sensitive to PD-Let plus radiotherapy treatment, and a dose-dependent decrease in survivability is seen. There seems also to be a dose-dependent response with respect to increased radiation. However, that response is less demarcated. The overall effect noted from the treatment indicates that bioprinting is causing the TIB cells to become more resistant to low doses of PD treatment and hence is a treatment to potentially avoid.

Increased resistance after TIB can potentially be generalized, as the TIB treatment of MCF7 breast cancer cells also rendered them more resistant than the MS cells. That resistance also manifested itself when followed by 10 Gy radiation. Only at the highest radiation tested here did the increased resistance of the TIB cells disappear. This effect was expected because palbociclib halts cell division at the G1 phase, then radiation exposure thoroughly decimates the cells that were fixed in this stage, thus responding to RT [15,22,26]. For the non-cancerous MCF-10A cells, there may be a slight increase in resistance for low drug concentrations and no radiation, but mostly, there is no increased resistance to the combinations of drugs and radiation treatments tested (see overall summary in Table 11). This behavior may be explained by the fact that bioprinting hyper-phosphorylates the cells and induces upregulation of genes associated with cell survival, as observed in our previous analysis [11].

In previous studies, we learned that bioprinting causes activation of critical kinases and chaperone proteins, e.g., HSP27 and HSP70, as it was observed in the phospho-MAPK array assay [11]. Additionally, TIB MCF7 BCCs significantly upregulates genes associated with cell survival and with stem cell properties, such as LUCAT1, CYP1A1, and others. These gene expressions may explain the response observed here [11]. LUCAT1 is a protein found in breast cancer cells with stem cell properties, and it has been linked to cell survival, and CYP1A1 has been associated with carcinogenesis [31,32]. It is possible that some cells possessing cancer stem-like properties were already present though in small quantities within the preprinted cell population. Hence, when these samples were bioprinted, most of the cells with non-stem cell characteristics perished due to the bioprinting process; thus, the remaining live cells, left to grow and divide, may have been those with stem cell phenotype, thus culminating in a higher number of cells with stem cell attributes.

The observed results indicate that the TIB cells could serve as an improved in-vitro tumor model, as the overall trend of these cells displayed enhanced survival. To develop new drugs, one would prefer to have more resilient cells. In addition, in-vivo responses to drugs and radiation can be similar to what we see in bioprinted cells, with some cells developing resistance and long-term survival. Actual breast cancer patients may present a similar response where they may develop drug resistance at specific drug dosages, displaying the hormesis effect, for example [21]. In the TIB MDA-MB-231 BCCs, a similar trend as the TIB MCF7 was detected. Higher viability was recorded in the bioprinted samples when radiation was omitted; this may be the desired outcome because bioprinting may elicit some immunity to drug treatments similar to in-vivo environments. Initially, we thought that bioprinting might have induced DNA damage, which enabled cells to become more biologically resistant to drugs. We have shown that bioprinting induced massive phosphorylation and protein expression [11], which may also stimulate MCF7 cells to become more active, resulting in more resistant cells.

This bioprinting approach to develop tumor models has not been tested with any other treatment combinations as performed here. Furthermore, to our knowledge, no quick drug-screening assay exists that uses thermal inkjet bioprinted breast cancer cells for drug discovery. Autologous biopsy samples could be processed and bioprinted to test appropriate protocol treatments to determine whether a course of medications will be beneficial for the patient. Personalized chemotherapy treatments are acquiring more momentum, and results are encouraging; yet, approved testing methods are in their infancy, resulting in a painfully slow process in clinical settings.

## 5. Conclusions

The present study evaluated the cell viability of human breast cancer cells utilizing a thermal inkjet printer to bioprint cells and compared them to manually seeded cells. We tested the synergistic effect when treated with the drug combination of the CDK 4/6 inhibitor palbociclib and the aromatase inhibitor letrozole applied concomitantly with radiation therapy. We aimed to prove whether the bioprinting process can be used to model breast tumors that can be used for drug discovery. We reported that TIB MCF7 cells have higher viability than the MS cells at lower concentrations of PD and RT. It seems that bioprinting is causing a hormesis effect on these cells, as previously distinguished [14]. Additionally, this work suggests that the combining these treatments given in parallel may help patients with luminal characteristics. For the TIB MDA-MB-231 cells, this treatment combination was proven ineffective at low concentrations of PD-Let because these cells resumed proliferation once RT was discontinued. As mentioned above, these cells lack Rb expression.

Future studies with 3D models utilizing this bioprinting technique should be explored in a separate investigation. In addition, RNA analysis of these cells post treatment is also highly suggested since RNA evaluation of post-printed cells presented in a previous publication resulted in substantial differences between the two sample types, TIB and MS. While this research was mainly conducted to compare the cell viability of the two cell-manipulation techniques, when treated with PD-Let given concomitantly with radiation therapy, these results can also be used as a guide for further studies with more complex tumor models made using these bioprinting technologies. Further analysis could be performed in bioprinted cells treated with these drug combinations plus radiotherapy to elucidate the molecular response and how cell signaling is affected. Foresights of a world where an autologous biopsy or blood samples of newly diagnosed breast cancer patients can be bioprinted and treated with their designated drug protocols and establishing whether a protocol will be effective in their treatment beforehand would be promising. Based on autologous bioprinted models, we anticipate that personalized therapies will provide better in-vitro models to predict if the assigned treatment will be useful before initiating such treatment regimen with a patient. We anticipate that a breast tumor model composed of TIB breast cancer cells will generate more accurate representations of tumor environments found in vivo.

## Figures and Tables

**Table 1:** CC_50_ values of Palbociclib for all three cell lines, MCF7, MDA-MB-231, and MCF-10A cells, and as a function of applied radiation. CC_50_ values obtained are for manually seeded (MS) and thermal inkjet bioprinted (TIB) cells without radiation exposure and at a radiation of 10 and 20 Gray.

Radiation	MS	TIB
PD CC_50_ values for MCF7 cells		
0 Gy	8 µM	30 µM
10 Gy	23 µM	40 µM
20 Gy	22 µM	23 µM
PD CC_50_ values for MDA-MB-231 cells		
0 Gy	41 µM	43 µM
10 Gy	36 µM	39 µM
20 Gy	8 µM	15 µM
PD CC_50_ values for MCF-10 A cells		
0 Gy	34 µM	40 µM
10 Gy	75 µM	80 µM
20 Gy	29 µM	31 µM

**Table 2:** Two-sample *t*-test results from the cell viability of TIB and MS, manually seeded, MCF7 luminal breast cancer cells when treated with PD-Let. No RT was applied.

PD Concentration Let = 10 µM	Mean % of Live Cells TIB	Mean % of Live Cells MS	Difference	95% CI	T-Value	*p*-Value
10 µM	96.8	89.5	7.3	(5.3, 9.2)	7.7	**<0.001**
50 µM	47.0	34.5	12.5	(4.7, 20.4)	3.4	**0.004**
100 µM	13.9	5.4	8.5	(0.9, 16.1)	2.3	**0.029**
H_2_O_2_	0.6	0.4	0.2	(7.8, 32.4)	0.6	0.545
Media	97.7	92.3	5.4	0.3, 10.5)	2.7	**0.042**
DMSO	93.5	98.4	−5.0	(−23.0, 13.0)	−1.2	0.355

**Table 3:** Two-sample *t*-test results from the cell viability of TIB and MS MCF7 luminal breast cancer cells when treated with PD-Let and RT = 10 gray applied in 3 fractions.

PD Concentration Let = 10 µM	Mean % of Live Cells TIB	Mean % of Live Cells MS	Difference	95% CI	T-Value	*p*-Value
10 µM	87.1	60.2	26.9	(19.0, 34.7)	8.1	**0.0001**
50 µM	38.1	29.6	8.5	(1.3, 15.6)	2.9	**0.027**
100 µM	14.2	18.0	−3.8	(−7.8, 0.2)	−2.2	0.061
H_2_O_2_	12.1	15.4	−3.3	(−16.8, 10.2)	−0. 8	0.494
Media	81.1	64.6	16.6	(−10.9, 44.1)	2.6	0.123
DMSO	84.0	68.9	15.1	(−10.6, 40.8)	2.5	0.127

**Table 4:** Two-sample *t*-test results from the cell viability of TIB and MS MCF7 luminal breast cancer cells when treated with PD-Let and RT = 20 gray applied in 5 fractions.

PD Concentration Let = 10 µM	Mean % of Live Cells TIB	Mean % of Live Cells MS	Difference	95% CI	T-Value	*p*-Value
10 µM	71.5	67.4	4.1	(−17.9, 26.1)	0.5	0.654
50 µM	5.4	7.6	−2.2	(−8.7, 4.2)	−0.9	0.419
H_2_O_2_	0.0	0.3	0.08	(−0.5, 0.01)	−3.0	0.057
DMEM	17.6	36.6	−18.9	(−92.6, 54.7)	−1.1	0.384
DMSO	83.7	84.6	−1.0	(−29.0, 27.2)	−0.1	0.898

**Table 5:** Two-sample *t*-test results from the cell viability of TIB and MS MDA-MB-231 cells when treated with palbociclib and letrozole without RT.

	Two-Sample *t*-Tests, MDA-MB-231 Cells, RT= 0 | H_0_: μ_1_ − µ_2_ = 0, H_1_: μ_1_ − µ_2_ ≠ 0						
PD Concentration Let = 10 µM	Mean %of Live Cells TIB	Mean % of Live Cells MS	Difference	95% CI (Lower-Upper)		T-Value	*p*-Value (2-Tailed)
10 µM	93.3	86.6	6.7	3.6	9.8	7.0	**0.004**
50 µM	40.7	39.6	1.1	−14.8	16.9	0.2	0.850
100 µM	1.5	0.9	0.6	0.4	0.8	8.9	**0.003**
150 µM	1.7	0.0	1.7	−0.9	4.4	2.1	0.13
Media	86.8	78.9	7.9	−6.7	22.4	1.7	0.18
DMSO	94.6	94.7	−0.2	−4.0	3.7	−0.2	0.88

**Table 6:** Two-sample *t*-test results from the cell viability of TIB and MS MDA-MB-231 cells when treated with PD-Let and radiation = 10 Gray in 3 fractions.

PD Concentration Let = 10 µM	Two-Sample *t*-Tests, MDA-MB-231 Cells, RT = 20 Gy | H_0_: μ_1_ − µ_2_ = 0, H_1_: μ_1_ − µ_2_ ≠ 0					
	Mean %of Live Cells TIB	Mean % of Live Cells MS	Difference	95% CI (Lower-Upper)	T-Value	*p*-Value
10 µM	57.0	42.0	15	(0.8, 29.1)	2.7	**0.04**
50 µM	1.8	15.8	−14	(−19.2, −8.8)	−7.5	**0.002**
100 µM	0.6	1.8	−1.3	(−2.2, −0.4)	−3.9	**0.018**
150 µM	0.1	1.4	−1.3	(−2.4, −0.1)	−3.0	**0.038**
H_2_O_2_	0.0	0.4	−0.4	(−0.6, −0.1)	−3.4	**0.042**
DMEM	8.1	59.6	−51.4	(−91.0, −11.9)	−5.6	**0.031**
DMSO	80.6	42.9	37.7	(−27.2, 100)	2.5	0.13

**Table 7:** Two-sample *t*-test results from the cell viability of TIB and MS MDA-MB-231 breast cancer cells when treated with PD-Let and RT = 20 gray in 5 fractions.

	Two-Sample *t*-Tests, MDA-MB-231 cells RT= 10 Gy | H_0_: μ_1_ − µ_2_ = 0, H_1_: μ_1_ − µ_2_ ≠0						
PD Concentration Let = 10 µM	Mean % of Live Cells TIB	Mean % of Live Cells MS	Difference	95% CI (Lower-Upper)		T-Value	*p*-Value (2-Tailed)
10 µM	91.3	96.9	−5.7	−8.4	−2.9	−5.8	**0.004**
50 µM	12.5	23.6	−11.2	−13.7	−8.7	−12.4	**<0.0001**
100 µM	5.9	5.1	0.8	−6.6	8.2	0.3	0.78
150 µM	0.1	0.5	−0.4	−1.3	0.5	−1.2	0.3
Media	85.6	93.7	−8.1	−16.2	0.1	−4.3	**0.05**
DMSO	95.9	99.1	−3.2	−6.3	−0.1	−4.4	**0.05**

**Table 8:** Two-sample *t*-test results from the cell viability of TIB and MS MCF-10A breast cells when treated with palbociclib + letrozole in conjunction without RT.

Two-Sample *t*-Tests, MCF-10A, RT= 0					H_0_: μ_1_ − µ_2_ = 0	H_1_: μ_1_ − µ_2_ ≠ 0
PD Concentration Let = 10 µm	Mean % of Live Cells TIB	Mean % of Live Cells MS	Difference	95% CI	T-Value	*p*-Value
10 µM	97.9	83.0	14.9	(2.5, 27.1)	3.8	**0.03**
50 µM	34.6	27.2	7.4	(−38.2, 53.1)	0.7	0.55
100 µM	0.3	15.1	−14.7	(−29.8, 0.3)	−4.2	**0.05**
150 µM	0.9	2.1	−1.2	(−5.0, 2.7)	−1.3	0.32
H_2_O_2_	0.0	0.00	−0.01	(−0.0, 0.0)	−2.0	0.18
Media	98.1	98.7	−0.6	(−15.1, 19.3)	−2.5	0.09
DMSO	96.9	94.8	2.1	(−1.5, 0.2)	0.5	0.65

**Table 9:** Two-sample *t*-test results from the cell viability of TIB and MS MCF-10A breast cells when treated with PD-Let in conjunction with RT of 10 Gy applied in 3 fractions.

PD Concentration Let = 10 µM	Two-Sample *t*-Tests, MCF-10A, RT= 10 gray				H₀: μ₁ − µ₂ = 0	H₁: μ₁ − µ₂ ≠ 0
	Mean % of Live Cells TIB	Mean % of Live Cells TIB	Difference	95% CI	T-Value	*p*-Value
10 µM	99.3	99.5	−0.1	(−0.8, 0.5)	−0.5	0.61
50 µM	71.0	69.1	1.9	(−0.9, 4.8)	1.6	0.15
100 µM	0.2	0.3	−0.04	(−0.2, 0.2)	−0.4	0.67
150 µM	0.0	0.0	−0.01	(−0.1, 0.1)	−0.5	0.65
H_2_O_2_	2.9	2.8	0.1	(−1.8, 2.0)	0.2	0.88
Media	99.6	99.0	0.6	(−0.1, 1.3)	3.4	0.07
DMSO	97.2	97.6	−0.5	(−1.1, 0.1)	−3.4	0.08

**Table 10:** Two-sample *t*-tests results from the cell viability of TIB and MS MCF-10A breast cells when treated with PD-Let in conjunction with RT of 20 Gy applied in 5 fractions.

PD Conc. Let = 10 µM	Two-Sample *t*-Tests, MCF-10A, RT= 20 Gray				H₀: μ₁ − µ₂ = 0	H₁: μ₁ − µ₂ ≠ 0
	Mean % of Live Cells TIB	Mean % of Live Cells MS	Difference	95% CI	T-Value	*p*-Value
10 µM	83.3	82.5	0.91	(−7.2, 8.9)	0.3	0.79
50 µM	18.8	15.4	3.4	(−3.8, 10.6)	1.1	0.30
H_2_O_2_	0.0	1.9	−1.9	(−7.4, 3.5)	−1.1	0.34
Media	86.4	83.6	2.9	(−14.0, 23.6)	0.5	0.66
DMSO	80.3	72.8	7.5	(−9.1, 25.5)	2.0	0.13

**Table 11:** Overall summary of findings.

Summary of TIB Compared to MS MCF7 Cell Samples	
With no RT	Slightly higher viability for all drug concentrations
With 10 Gray (Gy)	Slightly higher viability for lower drug concentrations
With 20 Gy	Not significant viability differences
Conclusion: TIB MCF7 cells at low PD-Let and RT dosages have a slightly higher viability than MS cells. Higher drug and RT dosages will kill all cells.	
Summary of TIB compared to MS MDA-MB-231 cell samples	
With no RT	Slightly higher viability at 10 μM of PD-Let concentrations
With 10 Gy	Less viability for lower drug concentrations
With 20 Gy	Higher viability for lowest drug concentration, lower viability for other drug concentrations
Conclusion: TIB MDA-MB-231 cell samples have higher viability than MS cells at the lowest drug concentrations. Radiation killed most of the cells at the two highest drug concentration but not at lower drug levels.	
Summary of TIB MCF-10A cells compared to MS samples.	
With no RT	Higher viability for low drug concentrations
With 10 Gy	Similar viability for all drug concentrations
With 20 Gy	Similar viability for all drug concentrations

## Data Availability

Supplemental data can be requested by email to: tboland@utep.edu.
